# Viscolin Inhibits *In Vitro* Smooth Muscle Cell Proliferation and Migration and Neointimal Hyperplasia *In Vivo*

**DOI:** 10.1371/journal.pone.0168092

**Published:** 2016-12-15

**Authors:** Chin-Chuan Chen, Chan-Jung Liang, Yann-Lii Leu, Yuh-Lien Chen, Shu-Huei Wang

**Affiliations:** 1 Graduate Institute of Natural Products, Chang Gung University, Taoyuan, Taiwan; 2 Chinese Herbal Medicine Research Team, Healthy Aging Research Center, Chang Gung University, Taoyuan, Taiwan; 3 Tissue Bank, Chang Gung Memorial Hospital, Taoyuan, Taiwan; 4 Center for Lipid and Glycomedicine Research (CLGR), Kaohsiung Medical University, Kaohsiung, Taiwan; 5 Center for Lipid Biosciences (CLB), Kaohsiung Medical University Hospital, Kaohsiung, Taiwan; 6 Center for Traditional Chinese Medicine, Chang Gung Memorial Hospital, Taoyuan, Taiwan; 7 Department of Anatomy and Cell Biology, College of Medicine, National Taiwan University, Taipei, Taiwan; Duke University, UNITED STATES

## Abstract

Viscolin, an extract of *Viscum coloratum*, has anti-inflammatory and anti-proliferative properties against harmful stimuli. The aim of the study was to examine the anti-proliferative effects of viscolin on platelet derived growth factor-BB (PDGF)-treated human aortic smooth muscle cells (HASMCs) and identify the underlying mechanism responsible for these effects. Viscolin reduced the PDGF-BB-induced HASMC proliferation and migration *in vitro*; it also arrested HASMCs in the G0/G1 phase by decreasing the protein expression of Cyclin D1, CDK2, Cyclin E, CDK4, and p21^Cip1^ as detected by Western blot analysis. These effects may be mediated by reduced PDGF-induced phosphorylation of ERK1/2, JNK, and P38, but not AKT as well as inhibition of PDGF-mediated nuclear factor (NF)-κB p65 and activator protein 1 (AP-1)/c-fos activation. Furthermore, viscolin pre-treatment significantly reduced neointimal hyperplasia of an endothelial-denuded femoral artery *in vivo*. Taken together, viscolin attenuated PDGF–BB-induced HASMC proliferation *in vitro* and reduced neointimal hyperplasia *in vivo*. Thus, viscolin may represent a therapeutic candidate for the prevention and treatment of vascular proliferative diseases.

## 1. Introduction

Vascular disease is characterized by chronic inflammation. In addition, vascular smooth muscle cell proliferation is a major event in atherosclerosis and restenosis progression. Therefore, modulation of inflammation and vascular smooth muscle cell (SMC) proliferation might have therapeutic effects for vascular disease[1].

Viscolin is a natural extract from *Viscum coloratum* used in the treatment of a wide range of diseases, including pleurisy, gout, arthritis, vascular disease, hypertension, inflammation and cancer[2]. In addition to its anti-inflammatory effects[3, 4], we have previously demonstrated that viscolin attenuates vascular cell adhesion molecule (VCAM) expression and monocyte adherence of tumor necrosis factor-α (TNF-α)-treated human umbilical vein endothelial cells (HUVECs)[5]. This protective effect appears critical in preventing the development of vascular inflammation and disease. Furthermore, viscolin exhibits anti-tumor activity, reducing tumor metastasis and inhibiting the proliferation of human hepatocarcinoma cells[6], human leukemia HL-60 cells, human myeloleukemic U937 cells[7–9], and carcinoma A253 cells[10]. However, it is unclear whether viscolin has a direct effect on cell cycle progression and the proliferation of vascular SMCs with potential to prevent highly proliferative vascular responses, such as post-angioplasty restenosis.

The aim of the study was to elucidate anti-proliferative effects of the viscolin on platelet derived growth factor-BB (PDGF-BB)-induced human aortic smooth muscle cells (HASMCs) and identify the mechanisms regulating these effects. In the present study, viscolin reduced PDGF-BB-induced proliferation of HASMCs by causing arrest in the G0/G1 phase and reducing the expression of PDGF-BB-induced cell cycle regulator proteins, including cyclin-CDK complexes and p21^Cip1^. Moreover, these inhibitory effects were mediated by decreased MAPK phosphorylation as well as nuclear factor (NF)-κB p65 and activator protein 1 (AP-1)/c-fos activation. Furthermore, pretreatment of viscolin significantly reduced the *in vivo* neointimal hyperplasia induced by endothelial-denudation.

## 2. Materials and Methods

### 2.1 Extraction and purification of viscolin

Viscolin, which was purified as described previously [2, 5, 11]. In brief, dried stems of *V*. *coloratum* nakai (family Loranthaceae) (471.0 g) were extracted with methanol, and combined methanol extracts were evaporated and partitioned to yield chloroform and aqueous extracts. The chloroform extract (10.5 g) was subjected to column chromatography over silica gel and eluted with chloroform and methanol step gradients to obtain 4.7 g of the active extract, PPE-SVC (CHCl_3_:MeOH = 9:1). PPE-SVC was subjected to chromatography once more on a silica gel column and eluted with a gradient of *n*-hexane and acetone to yield 53.6 mg of a new chalcone derivative, viscolin.

### 2.2 HASMC cultures

HASMCs, purchased as cryopreserved tertiary cultures (Cascade Biologics Inc., Portland, OR, USA), were grown in culture flasks in smooth muscle cell growth medium (M231, Cascade Biologics Inc.) supplemented with 5% smooth muscle growth supplement (Cascade Biologics Inc.). The growth medium was changed every other day until confluence, and then the cells were passaged every 3–5 days. Cells between passages 3 and 8 were used for the subsequent experiments. Before conducting experiments, HASMCs were pre-cultured in serum-starved medium (M231 alone) for 24 h.

### 2.3 Smooth muscle cell wound injury repair assay

HASMCs were grown in 6 cm culture dishes. After the cells were treated with 40 μM viscolin for 24 h, wounds were inflicted by dragging a sterile pipette tip across the monolayer, creating a 250 μm cell-free path after which 30 ng/mL of PDGF-BB (Pepro Tech, Rocky Hill, NJ, USA) was added. The area of cells grew into the wound area was determined after 24 h.

### 2.4 BrdU incorporation assay

HASMCs (5×10^4^) were cultured on gelatin-coated coverslips and were cultured in serum-starved medium for 24 h. After a pretreatment with viscolin for 24 h, 30 ng/mL of PDGF-BB and 10 mg/mL of 5-bromo-20-deoxyuridine (BrdU, Sigma-Aldrich, St. Louis, MO, USA) were added for another 24 h. The treated cells were fixed with a 95% ethanol/5% acetic acid solution for 30 min and were treated with 1N hydrogen chloride (HCl) for 10 min after which sodium borate was added to neutralize the HCl. The cells were next incubated with an anti-BrdU antibody (1:100; Sigma-Aldrich) at 4°C overnight followed by a FITC-conjugated goat anti-mouse IgG(1:200; Jackson ImmunoResearch, West Grove, Pennsylvania, USA). The cells were counterstained with 1 μg/mL DAPI (Sigma-Aldrich) and observed under a fluorescence microscope. Six fields were counted under a 20× objective lens to determine the number the number of BrdU-positive nuclei and total number of nuclei (DAPI-positive).

### 2.5 Cell cycle analysis

HASMCs were grown in 6-well culture plates and then serum-starved as described above. After pretreatment with 40 μM viscolin for 24 h and addition 30 ng/mL of PDGF-BB for another 1 h, the cells were trypsinization and stained with propidium iodide (PI). The cell-cycle progression was detected on a FACScan cytometer (BD Biosciences, San Jose, CA) and was analyzed by ModFit software.

### 2.6 Immunofluorescence analysis

HASMCs were cultured on glass coverslips and serum-starved as described above. After pretreatment with 40 μM viscolin for 24 h and addition 30 ng/mL of PDGF-BB for another 1 h, the cells were fixed with 4% paraformaldehyde for 15 min and then permeabilized with 0.05% Triton X-100 for 15 min. The fixed cells were blocked in 10% normal goat serum (Invitrogen, Carlsbad, CA, USA) for 1 h, and then incubated with rabbit anti-human p65 and c-fos antibodies (1:100 in blocking solution; GeneTex, Inc., San Antonio, TX, USA) at 4°C overnight. The cells were next incubated with FITC-conjugated goat anti-mouse IgG (1:200; Jackson ImmunoResearch) for 1 h at room temperature and then observed under a fluorescence microscope.

### 2.7 Immunoblotting analysis

HASMCs (4×10^6^) were cultured in 12-well plate. Serum-starved HASMCs were pretreated with 10, 20, 30, or 40 μM of viscolin for 24 h and then were incubated with 30 ng/mL PDGF-BB for different indicated time. The control group included cells in serum-free medium without viscolin or PDGF-BB. The cells were lysed with RIPA lysis buffer (Cell Signaling, Beverly, MA, USA), and 20 μg of protein was separated by 10–12% SDS–PAGE and transferred onto PVDF membranes (Millipore, Bedford, MA, USA). After blocking with 5% BSA at room temperature for 1 h, the membranes were incubated with rabbit anti-human cyclin D1, CDK2, p21^Cip1^, p27^Kip1^ antibodies (all 1:1000 in 1.5% BSA; GeneTex); mouse anti-human cyclin E and CDK4 antibodies (both 1:1000 in 1.5% BSA; Cell Signaling); rabbit anti-human phospho-P38, phospho-ERK, phospho-JNK, c-fos (all 1:1000 in 1.5% BSA; Santa Cruz Biotechnology); and rabbit anti-human p65, anti-human phospho-AKT (all 1:1000 in 1.5% BSA; GeneTex) at 4°C overnight. The membranes were next incubated with HRP-conjugated anti-mouse IgG antibodies or anti-rabbit IgG (all 1:3000 in PBS; GeneTex) at room temperature for 1 h. Immunoreactivity was detected with ECL (GE Healthcare Bioscience, Piscataway, NJ, USA). The intensities of the bands were quantified using Gel-Pro software (MediaCybernetics, Rockville, MD, USA). Rabbit anti-human GAPDH, α–tubulin and β–actin, Histon and lamin B1 antibodies were used as an internal control (all 1:3000 in 1.5% BSA; GeneTex). The intensities of the target proteins were normalized by the intensities of the internal control bands.

### 2.8 Mouse femoral injury model and viscolin treatment

Male C57BL/6J mice (8 weeks-old) were purchased from the National Laboratory Animal Center (Taipei, Taiwan). All procedures were performed in accordance with the local institutional guidelines for animal care of the National Taiwan University and complied with the “Guide for the Care and Use of Laboratory Animals” NIH publication No. 86–23, revised 2011. The protocol was approved by the National Taiwan University College of Medicine and College of Public Health Institutional Animal Care and Use Committee (IACUC NO: 20150293). Surgical procedures were performed as described by Sata et al [12]. In brief, the mice were anesthetized by intraperitoneal injection of 70 mg/kg body weight of pentobarbital, and endothelial denudation of the femoral artery was undertaken by transluminal mechanical injury. The mice were divided into the following two treatment groups: (1) 50 μL of PBS as a control and (2) 100 μg/kg body weight of viscolin in 50 μL of PBS. Treatments were administered by intraperitoneal injection on days 0–1 before the injury. The mice were sacrificed by an overdose of pentobarbital 14 days after the trans-luminal mechanical injury. The left femoral artery was gently dissected, fixed in 4% paraformaldehyde, embedded in OCT, and cross-sectioned for morphometric analysis and immunohistochemistry. The 5-mm femoral artery was cut serially into 8 μm frozen sections, and every tenth section was stained with a Resorcin-Fuchsin solution (Sigma-Aldrich). For morphometric analysis of neointimal formation, the mean neointima/media cross-sectional area (I/M) ratio was detected as described previously [13].

### 2.9 TUNEL assay

TUNEL assay was used to detect DNA fragmentation of the cells *in situ* according to the instructions provided by the manufacturer (Roche, Applied Science, Germany). Briefly, adherent cultured cells were fixed in 4% paraformaldehyde for 15 min at room temperature, and then incubated with the 0.1% triton X-100 in PBS for 15 min at room temperature. After washing in PBS, the cells were incubated with terminal deoxynucleotidyl transferase (TdT) and a mixture of fluorescent-labeled nucleotides for 60 min at 37°C, counterstained with DAPI and observed by florescence microscopy. For positive controls (PC), cells treated with 1 mg/mL of DNase I at room temperature for 20 min.

### 2.10 Knockdown of gene expression

Knockdown of JNK, ERK and P38 gene expression was performed by transfection with small interfering RNA (siRNA). HASMCs (5×10^6^) were incubated in 100 μL of nucleofector solution (Lonza, Allendale, NJ, USA), and gene-specific siRNA oligomers (1 μM; Invitrogen) were electroporated according to the manufacturer’s instructions. Cells were transfected for 48 h after which protein expression was evaluated by Western blot analysis.

### 2.11 Statistical analysis

All values are provided as mean±SEM. Statistical comparisons were made using the Student’s t-test and one-way ANOVA. Significance was defined as *P-*values*<*0.05.

## 3. Results

### 3.1 Viscolin suppressed PDGF-BB-induced HASMC proliferation and migration

**Fig 1:**
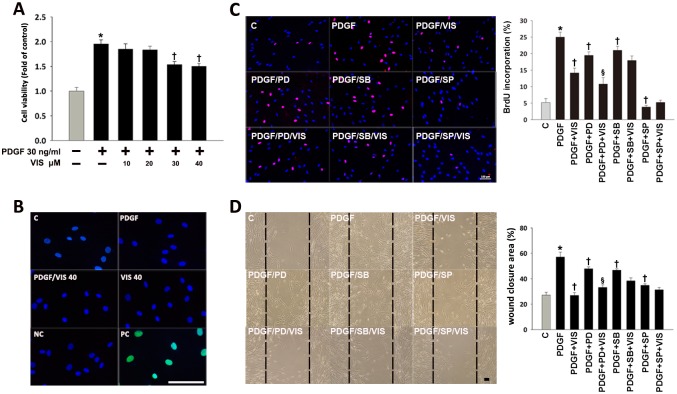
Viscolin reduces the migration and proliferation of PDGF-BB-treated HASMCs. Serum-starved HASMCs were cultured with PDGF-BB (PDGF) alone or with viscolin (VIS). (A) Cell viability was measured using the MTT assay. (B) Cell death was assessed using the TUNEL assay. Cells treated with 1 mg/mL DNase I were the positive controls (PC). (C) BrdU incorporation was used to determine HASMC proliferation. Some cells were pretreated with 30 μM MAPK inhibitors for 30 min prior to treatment with viscolin or PDGF-BB. Representative photographs are shown in the left panel, and the quantitative data relative to the control value (without any treatment) were shown in the right panel. (D) Cell migration was examined in a wound healing assay. Serum-starved HASMCs were wounded by scratch injury (black lines on Fig). The wound closure area was determined by measuring the wound area/initial wound area. Before wound formation and PDGF-BB and or/ viscolin treatment, some cells were pretreated with an ERK1/2 inhibitor (PD98059; PD), P38 inhibitor (SB203580; SB) or JNK inhibitor (SP600125; SP) for 30 min. Representative inverted phase contrast light microscopy photographs are shown in the left panel and the quantified data are shown in the right panel. In A, C, and D, the data are means±SEM for four independent experiments. *P<0.05 vs. the untreated group; ^†^*P*<0.05 vs. the PDGF-BB-treated group; ^‡^*P*<0.05 vs. the viscolin/PDGF-BB group. The scale bars in B- D = 100 μm.

The proliferation and migration of vascular SMCs, which can be induced by PDGF-BB[14], play an important role in the vascular injury[1]. To examine the effects of viscolin on the proliferation and migration of PDGF-BB-treated HASMCs, MTT assay, BrdU incorporation and wound healing assays were performed. As shown in Fig 1A, cell viability increased with 30 ng/mL of PDGF-BB for 24 h as compared to control cells; however, these effects were significantly reduced by viscolin (30–40 μM) treatment (*P*<0.05). In addition, the BrdU-positive cells after treatment with PDGF-BB increased by 4.5-fold over the control group, which was significantly inhibited by viscolin pretreatment for 24 h (*P*<0.05; Fig 1C).To exclude the possibility of viscolin-induced toxicity, TUNEL assay was used. As shown in Fig 1B, neither PDGF-BB nor viscolin were toxic to HASMCs. Finally, viscolin significantly reduced PDGF-BB-induced HASMC migration as shown in an *in vitro* wound healing assay (*P*<0.05; Fig 1D). The re-endothelialization, endothelial protection and endothelial proliferation all play important roles in angioplasty. To examine the effects of viscolin on endothelial cell growth, crystal violet cell proliferation assay was performed. The cell growth of viscolin for HUVEC was, respectively, 1.00±0.01, 1.05±0.02, 1.02±0.01, 0.98±0.01, or 1.01±0.02 of control levels. The results showed that 0–40 μM viscolin was no effect on HUVEC growth. Taken together, viscolin can inhibit PDGF-BB-induced HASMC proliferation and migration with no effect to the endothelial cell growth.

### 3.2 G0/G1 cell cycle arrest of PDGF-BB-treated HASMCs by viscolin

**Fig 2:**
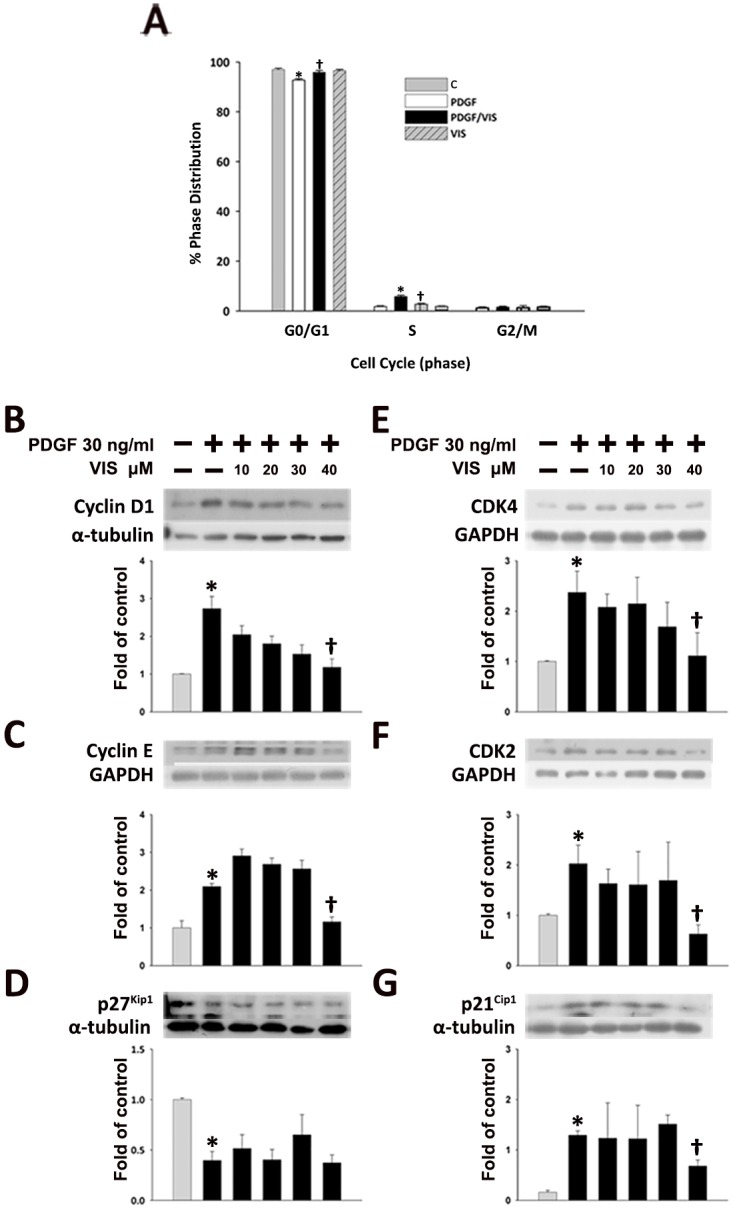
Viscolin induces G0/G1 arrest and alters the expression of cell cycle regulatory proteins in PDGF-BB-treated HASMCs. (A) Cells were left untreated or were treated with 40 μM viscolin for 24 h and then with PDGF-BB for an additional 24 h. The proportions of cells in the G0/G1, S, or G2/M phases were determined by FACScan analysis. The mean±SEM of three independent experiments were shown. *P<0.05 vs. the untreated group; ^†^*P*<0.05 vs. the PDGF-BB-treated group. (B-G) Serum-starved HASMCs were cultured in the presence of 10, 20, 30, or 40 μM viscolin for 24 h and then treated with 30 ng/mL of PDGF-BB for an additional 24 h. (B) The expression of Cyclin D1, (C) Cyclin E, (D) p27^Kip1^, (E) CDK4, (F) CDK2, (G) or p21^Cip1^ proteins was determined by Western blot analysis. GAPDH or α-tubulin was processed in parallel as an internal control for protein loading. The results are shown as the fold increase in expression relative to that in untreated controls. The data are the means ± SEM (n = 4). *P<0.05 vs. the untreated controls; ^†^*P*<0.05 vs. the PDGF-BB-treated cells.

To determine whether the effects of viscolin on HASMC growth was due to cell cycle arrest, flow cytometry was performed. As shown in Fig 2A and in S1 Table, HASMCs were all synchronized in the G0/G1 phase after serum starvation. With PDGF-BB stimulation, the proportion of HASMCs in S phase increased from 1.80±0.24% to 5.73±0.58%, which was reduced by viscolin pre-treatment (5.73±0.58% vs. 2.78±0.27%) and accompanied by a significant accumulation of cells in the G0/G1 phase from 92.74±0.62% to 95.92±0.74% (*P*<0.05). These data indicate that viscolin suppresses HASMC proliferation via G0/G1 arrest.

### 3.3 Viscolin-mediated arrest of PDGF-BB-treated HASMCs is associated with decreased protein expression of cyclins, CDKs and p21^cip1^

To determine the mechanisms underlying the inhibition of PDGF-BB-treated HASMC proliferation by viscolin, we examined the effect of the viscolin on cell cycle regulatory proteins, including cyclins and cyclin-dependent kinases (CDKs)[15, 16], by Western blot analysis. PDGF-BB treatment significantly increased the expression of Cyclin D1 (Fig 2B), Cyclin E (Fig 2C), CDK4 (Fig 2E), and CDK2 (Fig 2F), which was reduced by 40μM viscolin pre-treatment (*P*<0.05)

We also determined the effect of viscolin on p27^Kip1^ and p21^Cip1^, two cyclin-dependent kinase inhibitors (CDKIs). With PDGF-BB stimulation, p27^Kip1^ expression was significantly reduced (*P*<0.05) which was not influenced by viscolin treatment (Fig 2D). In contrast, p21^Cip1^ expression was increased with the PDGF-BB treatment, and was blocked by 40 μM viscolin pre-treatment (*P*<0.05; Fig 2G). Thus, viscolin reduced the proliferation of PDGF-BB-induced HASMCs by inhibiting the expression of cyclins/CDKs and p21^Cip1^.

### 3.4 Effects of viscolin on MAPKs in PDGF-BB-treated HASMCs

**Fig 3:**
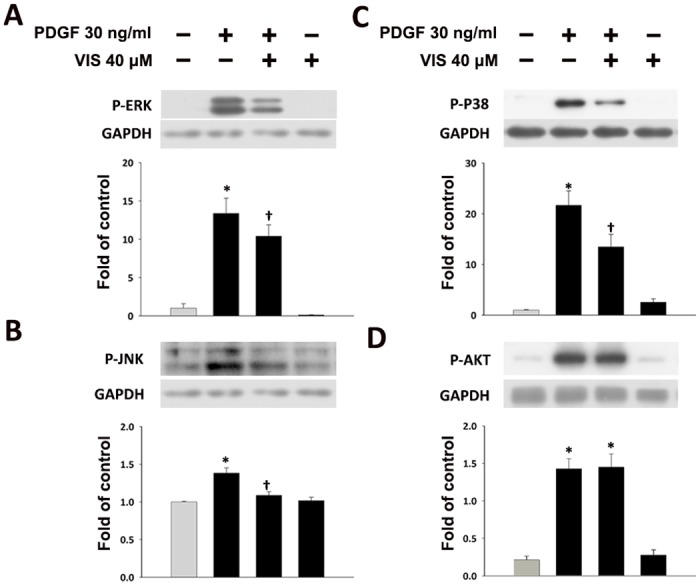
Effects of viscolin expression on the phosphorylation of MAPKs and AKT in PDGF-BB-treated HASMCs. Cells were treated with 40 μM viscolin for 24 h and then incubated with or without 30 ng/mL PDGF-BB for 30 min. Phosphorylated (A) JNK, (B) P38, (C) ERK1/2, or (D) AKT levels were determined by Western blot analysis. GAPDH was processed in parallel as an internal control for protein loading. The histograms show the phosphorylated band/GAPDH ratio for (A-D). *P<0.05 vs. the untreated control, ^†^*P*<0.05 vs. the PDGF-BB-treated cells.

Previous studies showed that MAPKs and AKT play an important role in cell proliferation [17]. Therefore, the effects of viscolin on the expression of MAPKs and AKT were determined in PDGF-BB-stimulated HASMCs. The phosphorylation of ERK (P-ERK, Fig 3A), JNK (P-JNK, Fig 3B) and P38 (P-P38, Fig 3C) was up-regulated by PDGF-BB treatment, which was significantly reduced by 40 μM viscolin pre-treatment (*P*<0.05). However, P-AKT expression was not altered by viscolin. Therefore, the effects of PDGF-BB and viscolin on HASMC cell cycle regulatory protein expression may be mediated by activation of MAPKs.

### 3.5 The effects of viscolin on the proliferation and migration of PDGF-BB-stimulated HASMCs are mediated by MAPK activation

To determine if MAPK activation was necessary for the effects of viscolin on DNA synthesis, HASMCs were treated for 1 h with 30 μM PD98059, an ERK1/2 inhibitor, SB203580, a P38 inhibitor, or SP600125, a JNK inhibitor, prior to analysis of BrdU incorporation. As shown in Fig 1C and 1D, PD98059, SB203580, and SP600125 significantly reduced PDGF-BB-induced BrdU incorporation as well as HASMC migration (*P*<0.05). Moreover, co-treatment with viscolin and PD98059 had additive effect on BrdU incorporation and HASMC migration as compared with PD98059 treatment alone, suggesting that the inhibition of PDGF-BB-treated HASMC proliferation and migration by viscolin was mainly mediated through the inhibition of P38 and JNK phosphorylation.

**Fig 4:**
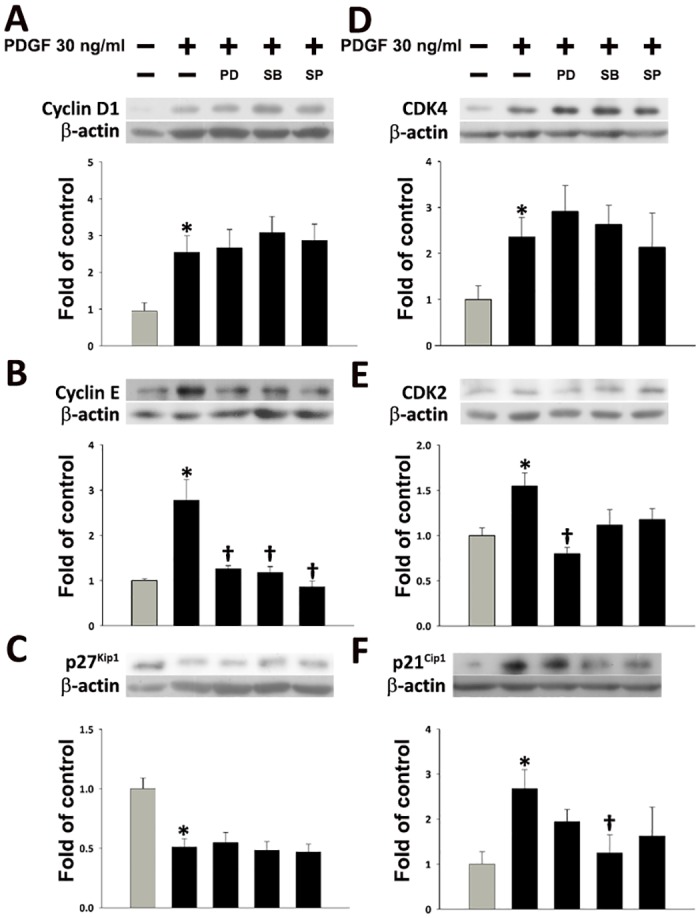
Effects of MAPK inhibitors on cell cycle regulator protein expression in PDGF-BB-treated HASMCs. Cells were treated with 30 μM MAPK inhibitors for 1 h, and then incubated with 30 ng/mL PDGF-BB for 24 h. The expression of (A) Cyclin D1, (B) Cyclin E, (C) p27^Kip1^, (D) CDK4, (E) CDK2, (F) or p21^Cip1^ proteins was determined by Western blot analysis. β-actin was processed in parallel as an internal control for protein loading. The histograms show the phosphorylated band/β-actin ratio (A-F). *P<0.05 vs. the untreated control, ^†^*P*<0.05 vs. the PDGF-BB-treated cells.

As shown in Fig 4, the expression of cyclin E, CDK2, and p21^Cip1^ was reduced in PDGF-BB-treated HASMCs upon pretreatment with inhibitors. Specifically, PDGF-BB-induced Cyclin E expression was inhibited by pretreatment with PD98059, SB203580, and SP600125 (*P*<0.05; Fig 4B). PDGF-BB-induced CDK2 expression was inhibited by PD98059 (*P*<0.05; Fig 4E), and PDGF-BB-mediated p21^Cip1^ expression was inhibited by SB203580 (*P*<0.05; Fig 4F). However, PDGF-BB-induced cyclin D1, CDK4, and p27^Kip1^ expression was not affected by any of the inhibitors analyzed (Fig 4A, 4C and 4D).

**Fig 5:**
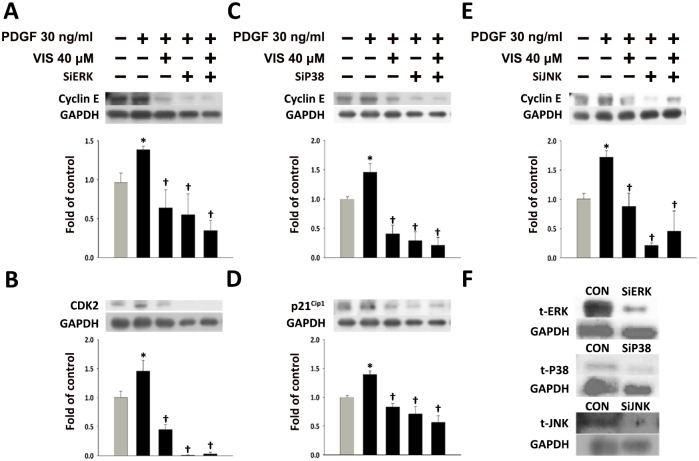
Effects of ERK, P38, and JNK-specific siRNA on cell cycle regulator protein expression in PDGF-BB-treated HASMCs. The expression of the (A) Cyclin D1, (B) CDK4, (C) Cyclin E, (D) CDK2, and (E) p21^Cip1^ proteins was determined by Western blot analysis after ERK, P38, and JNK silencing, viscolin and PDGF-BB treatment. (F) ERK, P38, and JNK-specific siRNAs reduced the total levels of the ERK, P38, and JNK proteins compared with the non-treated cells. The values are the means±SEM. *P<0.05 vs. the untreated control, ^†^*P*<0.05 vs. the PDGF-BB-treated cells.

To more fully evaluate the role of MAPKs in mediating the effects of viscolin, HASMCs were transfected with ERK-, P38-, or JNK-specific siRNA and were treated with PDGF-BB without and with viscolin. The effectiveness of each siRNA was confirmed by Western blot analysis for ERK, P38, and JNK protein expression (Fig 5F). As shown in Fig 5A, 5C and 5E, PDGF-BB-induced Cyclin E expression was reduced after transfection with ERK-, P38-, or JNK-specific siRNA (*P*<0.05). In addition, ERK- and P38-specific siRNA significantly inhibited the expression of CDK2 (Fig 5B) and p21^Cip1^ (Fig 5D), respectively. These results suggest that the reduction PDGF-BB-induced HASMC proliferation and migration by viscolin may be mediated through MAPK activation to regulate the cyclin-CDK and p21^Cip1^ expression.

### 3.6 Viscolin decreases NF-κB p65 and AP-1/c-fos nuclear translocation in PDGF-BB-treated HASMCs

**Fig 6:**
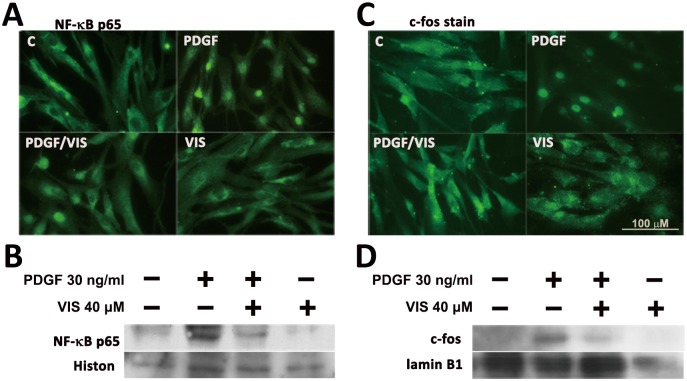
Effects of viscolin on NF-κB p65 and AP-1/c-fos nuclear translocation. HASMCs were pretreated with 40 μM viscolin for 24 h and then were treated with 30 ng/mL PDGF-BB for 30 min. Immunofluorescence staining or Western blot analysis were performed to show subcellular localization of (A, B) NF-κB p65 and (C, D) c-fos. Histone or lamin B1 was processed in parallel as an internal control for protein loading. Representative results from three separate experiments are shown. Bar = 100 μm.

Transcriptional regulation involving NF-κB and AP-1 activation has been implicated in cytokine-induced SMC proliferation and migration [18–20].Therefore, the effects of viscolin and PDGF-BB on NF-κB and AP-1 subcellular localization were also analyzed in HASMCs. HASMCs stimulated with PDGF-BB for 30 min exhibited stronger NF-κB p65 and AP-1/c-fos nuclear staining as compared to control cells (Fig 6A and 6C), which was reduced in viscolin-pretreated cells (PDGF-BB/VIS) that had stronger cytoplasmic staining. Consistent with the immunofluorescence analysis, the expression of NF-κB p65 and AP-1/ c-fos was greater in nuclear fraction with PDGF-BB treatment, and was reduced by viscolin pre-treatment (Fig 6B and 6D). These data suggest that viscolin inhibited HASMC proliferation induced by PDGF-BB at least in part through reducing the nuclear translocation of NF-κB p65 and AP-1/c-fos.

### 3.7 Viscolin reduces neointimal hyperplasia *in vivo*

**Fig 7:**
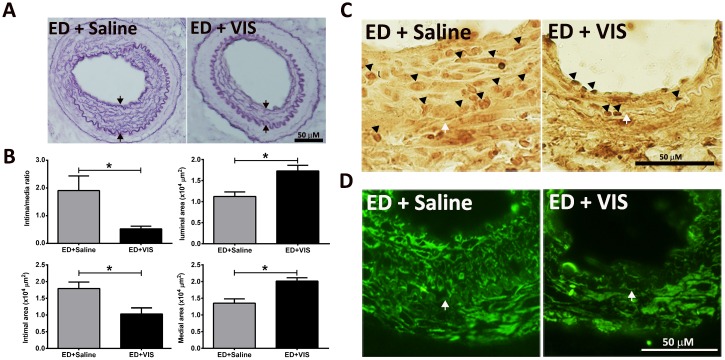
Viscolin reduces neointimal hyperplasia *in vivo*. (A) Representative cross-sections of injured femoral arteries from ED/saline-injected mice (left) and ED/viscolin-injected mice (right) were stained with Resorcin–Fuchsin. Neointimal hyperplasia was shown between two arrows. Bar = 50 μm. (B) Quantification of the neointima/media (I/M) area ratio, intimal area, luminal area, and medial area. The values are means±SEM. *P<0.05 vs. the ED/saline-injected mice. (C) Immunohistochemical staining with anti-PCNA antibodies. The positive reaction and internal elastic lamina are indicated by arrowheads and an arrow, respectively. Bar = 50 μm. (D) Immunohistochemical staining for the smooth muscle cell marker, α-actin. The internal elastic lamina is indicated by an arrow. Bar = 50 μm.

The effects of viscolin on neointimal formation was next evaluated after endothelial denudation (ED) of the femoral artery *in vivo*. As shown in Fig 7A, the histological analysis revealed that ED significantly increased the intimal/media ratio and intimal area, and viscolin significantly increased the luminal area and media area (*P*<0.05; Fig 7B). Analysis of PCNA and the SMC marker, α-actin, revealed more PCNA- and α-actin-positive cells in the thickened intima of the control (ED+Saline) group as compared to the intima of the ED+VIS group (Fig 7C and 7D).

## 4. Discussion

**Fig 8:**
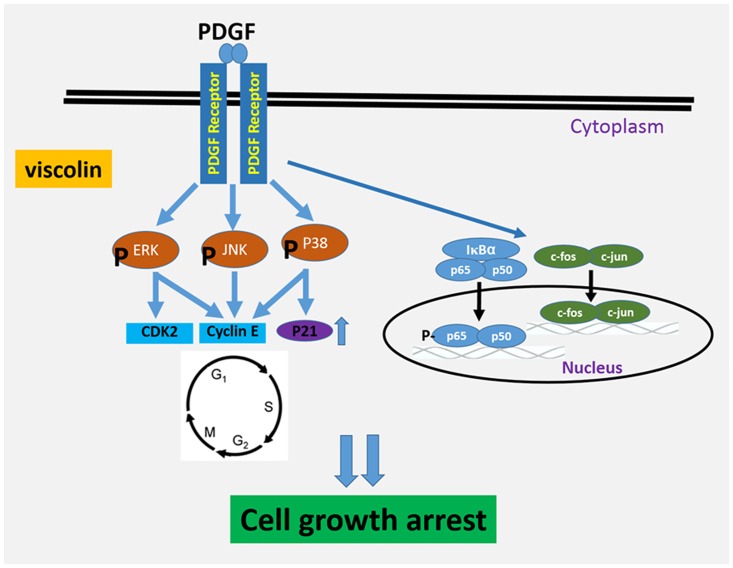
Graphical scheme of the anti-proliferative mechanism of viscolin in PDGF-treated HASMCs.

In the present study, viscolin significantly inhibited PDGF-BB-induced proliferation and migration of HASMCs *in vitro*. Viscolin arrested cells in the G0/G1 phase at least in part by reducing the expression of cell cycle-related proteins through the suppression of MAPK signaling and activation of NF-κB p65 and c-fos (Fig 8). In addition, viscolin attenuated neointimal hyperplasia induced by ED *in vivo*. Thus, a novel therapeutic effect of viscolin on vascular injury-related disease is presented.

Viscolin is a natural extract from *Viscum coloratum* which is a traditional Chinese medicine that is used to treat heart disease, atherosclerosis, inflammatory bowel disease, arthritis and hypertension given its anti-inflammatory and antioxidant properties [2]. Our previous report also demonstrated that viscolin can prevent TNF-α-induced HUVEC apoptosis and inflammation by exerting an antioxidant effect [5]. In addition, viscolin could reduce cancer cell growth and metastasis. Furthermore, Korean mistletoe (derived from a *V*. *coloratum* extract) induced apoptosis of human myeloleukemic U937 and hepatocarcinoma cells by activating caspase cascades [8] and inhibiting telomerase via a mitochondrial controlled pathway [21], respectively. To our knowledge, the present study is the first to show that viscolin suppressed PDGF-BB-induced HASMC proliferation by triggering G0/G1 phase arrest.

VSMCs normally exist in a quiescent state in the artery media. After injury or inflammatory cytokine stimulation, the abnormal proliferation and migration of vascular SMCs from the media into the intima induces neointimal hyperplasia[1]. In the present study, pretreatment with viscolin reduced PDGF-BB-mediated HASMC proliferation, delayed the transition from the G1 phase to the S phase of the cell cycle, and impaired migration.

Cell proliferation is regulated by cell cycle regulatory proteins, such as cyclins, CDKs and CDKIs. Cyclin D1/CDK4 and Cyclin E/CDK2 complexes are essential for entry into S phase and are negatively regulated by CDKIs, such as p21^Cip1^. In contrast, recent studies showed that p21^Cip1^ can act as a scaffold to facilitate the assembly of cyclins and CDKs required for DNA synthesis [22, 23]. Consistent with these studies, viscolin reduced the expression of Cyclin D1/CDK4, Cyclin E/CDK2 and p21^Cip1^ to exert its anti-proliferative properties, suggesting that viscolin can inhibit cell proliferation through the regulation of multiple targets.

Previous studies have shown that MAPKs play an important role in vascular SMC proliferation [24]. Consistent with previous studies, PDGF-BB activated three MAPKs in HASMCs [25]. In the present study, viscolin significantly reduced MAPK expression in PDGF-BB-induced HASMCs. The addition of MAPK inhibitors revealed that viscolin might primarily inhibit PDGF-BB-induced HASMC proliferation and migration mainly through the P38 and JNK pathways rather than through the ERK pathway. In addition, silencing of MAPKs suggested that the effects of viscolin on cell cycle-related proteins might be mediated through different pathways (e.g., cyclin E was regulated by all three MAPKs, CDK2 was regulated by ERK, and p21^Cip1^ was regulated by P38). Thus, multiple targets might mediate the effects of viscolin.

PDGF-BB is a potent mitogen of vascular SMCs and plays an important role in atherosclerosis and restenosis [14]. Recent studies have shown that it can induce vascular SMC proliferation through the activation of transcriptional factors, such as NF-κB and AP-1, that can be regulated by phosphorylation of MAPKs. In the present study, viscolin significantly inhibited the nuclear translocation of NF-κB p65 and AP-1/c-fos from the cytoplasm in the PDGF-BB-treated HAMSCs.

The re-endothelialization, endothelial protection and inhibition of VSMC proliferation all play important roles in angioplasty. Sirolimus has been shown to be effective at inhibiting neointimal hyperplasia by blocking VSMC proliferation and migration, and it is used on drug-eluting stents for percutaneous transluminal coronary angioplasty. However, the therapeutic limitations of sirolimus for angioplasty are the delayed re-endothelialization through endothelial cellular senescence via Sirtuin 1 down-regulation[26] or telomerase inactivation[27], reduced endothelial progenitor cell mobilization[28], and increased tissue factor activity that promotes arterial thrombosis[29]. Compared to the effect of sirolimus on endothelial cells, viscolin not only has no influence on cell viability and growth but also exerts anti-inflammatory and anti-oxidative properties in TNF-ɑ-treated endothelial cells [5]. Furthermore, Korean mistletoe (derived from a *V*. *coloratum* extract) significantly increases mitochondrial function[30], which contributes to a delay in the aging process[31]. These results indicated the viscolin has greater therapeutic potential for angioplasty.

In conclusion, viscolin inhibited the *in vitro* proliferation and migration in PDGF-BB-treated HAMSCs and femoral artery neointimal hyperplasia *in vivo*. The anti–proliferative effects of viscolin were mediated in part by regulating cell cycle-related regulatory proteins as well as inhibiting phosphorylation of MAPKs and activation of NF-κB and AP-1. Thus, viscolin may represent a therapeutic candidate for cardiovascular diseases.

## Supporting Information

S1 Table Viscolin Inhibits the Proliferation of PDGF-treated HASMCs by Causing Cell-Cycle Arrest at G0/G1 Phase. (TIF)
