# Supplementary figures and images for: Viscolin Inhibits In Vitro Smooth Muscle Cell Proliferation and Migration and Neointimal Hyperplasia In Vivo
Source: PLoS One. 2016 Dec 15;11(12):e0168092. doi: 10.1371/journal.pone.0168092 (PMC5158191; doi:10.1371/journal.pone.0168092)

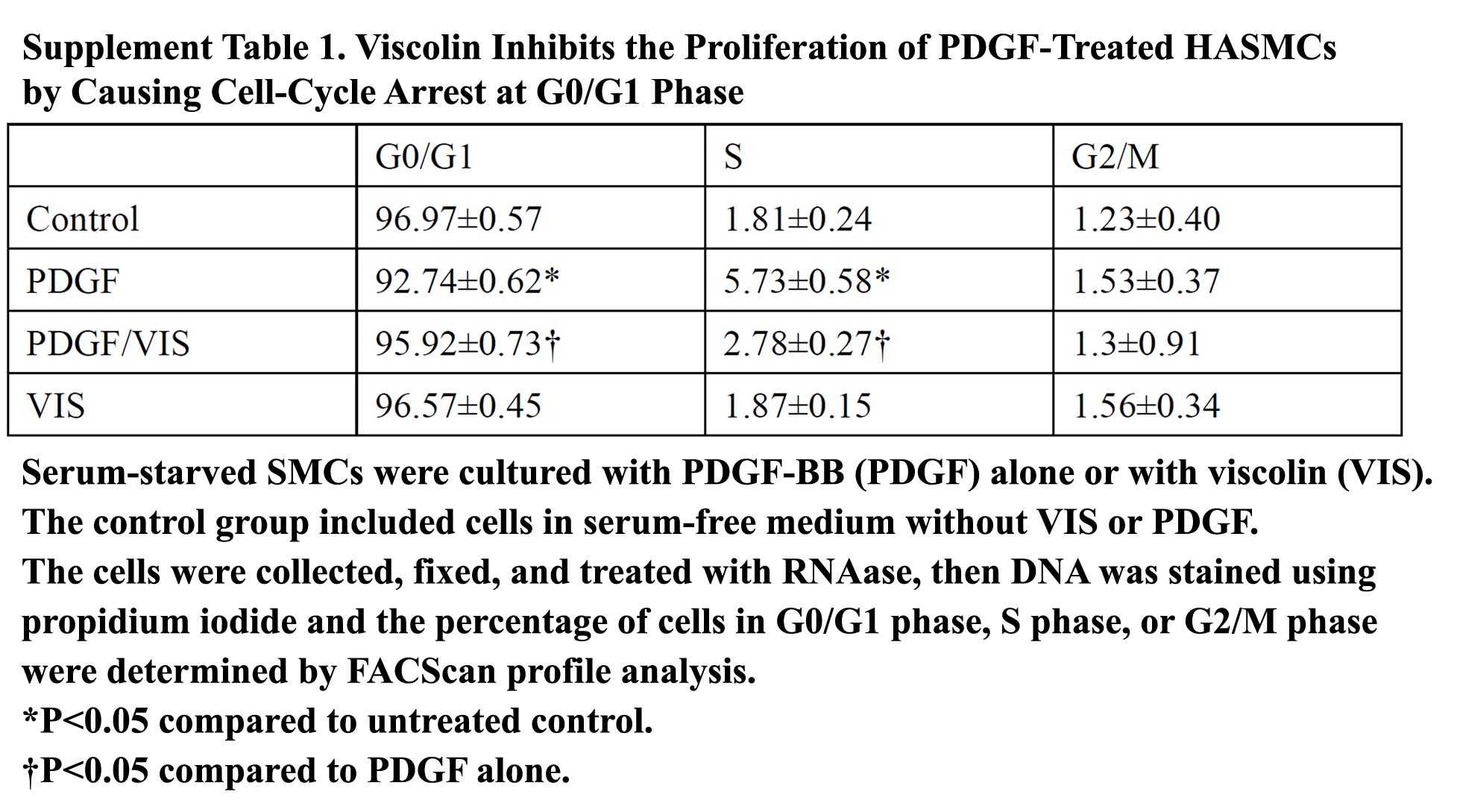

Supplement: S1 Table — (TIF) [file pone.0168092.s001.tif]
